# Machine Learning-based Individual Assessment of Cortical Atrophy Pattern in Alzheimer’s Disease Spectrum: Development of the Classifier and Longitudinal Evaluation

**DOI:** 10.1038/s41598-018-22277-x

**Published:** 2018-03-07

**Authors:** Jin San Lee, Changsoo Kim, Jeong-Hyeon Shin, Hanna Cho, Dae-seock Shin, Nakyoung Kim, Hee Jin Kim, Yeshin Kim, Samuel N. Lockhart, Duk L. Na, Sang Won Seo, Joon-Kyung Seong

**Affiliations:** 1 Department of Neurology, Samsung Medical Center, Sungkyunkwan University School of Medicine, Seoul, 06351 Korea; 2 Neuroscience Center, Samsung Medical Center, 06351 Seoul, Korea; 3 Department of Neurology, Kyung Hee University Hospital, Seoul, Korea; 4 Department of Preventive Medicine, Yonsei University College of Medicine, Seoul, South Korea; 5 Department of Bio-convergence Engineering, Korea University, Seoul, Korea; 6 School of Biomedical Engineering, Korea University, Seoul, Korea; 7 Department of Neurology, Gangnam Severance Hospital, Yonsei University College of Medicine, Seoul, Korea; 8 MIDAS Information Technology Co., Ltd, Seoul, Korea; 9 Helen Wills Neuroscience Institute, University of California Berkeley, Berkeley, CA 94720 USA; 10 Department of Internal Medicine, Division of Gerontology and Geriatric Medicine, Wake Forest School of Medicine, Winston-Salem, NC 27157 USA; 11 Department of Health Sciences and Technology, Sungkyunkwan University, Seoul, 06351 Korea; 12 Clinical Research Design and Evaluation, SAIHST, Sungkyunkwan University, Seoul, 06351 Korea

## Abstract

To develop a new method for measuring Alzheimer’s disease (AD)-specific similarity of cortical atrophy patterns at the individual-level, we employed an individual-level machine learning algorithm. A total of 869 cognitively normal (CN) individuals and 473 patients with probable AD dementia who underwent high-resolution 3T brain MRI were included. We propose a machine learning-based method for measuring the similarity of an individual subject’s cortical atrophy pattern with that of a representative AD patient cohort. In addition, we validated this similarity measure in two longitudinal cohorts consisting of 79 patients with amnestic-mild cognitive impairment (aMCI) and 27 patients with probable AD dementia. Surface-based morphometry classifier for discriminating AD from CN showed sensitivity and specificity values of 87.1% and 93.3%, respectively. In the longitudinal validation study, aMCI-converts had higher atrophy similarity at both baseline (*p* < 0.001) and first year visits (*p* < 0.001) relative to non-converters. Similarly, AD patients with faster decline had higher atrophy similarity than slower decliners at baseline (*p* = 0.042), first year (*p* = 0.028), and third year visits (*p* = 0.027). The AD-specific atrophy similarity measure is a novel approach for the prediction of dementia risk and for the evaluation of AD trajectories on an individual subject level.

## Introduction

Alzheimer’s disease (AD) dementia is the most common form of dementia^1^. Previous neuroimaging studies have shown that patients with AD demonstrate characteristic patterns of cortical atrophy at a group-level, especially in the medial temporal, temporoparietal, posterior cingulate, and precuneus regions^2,3^. Recent classification methods also provide a general framework to classify individual subjects, for example using arbitrary features defined on a three-dimensional (3D) cortical surface^4–7^ or volumetric features^8–10^. These classification methods have demonstrated adequate performance in an individual subject analysis with high accuracy. Both group- and individual-level analyses have successfully demonstrated the discriminating power of cortical atrophy patterns for AD diagnosis.

Amnestic mild cognitive impairment (aMCI) refers to a transitional state between normal cognition and dementia. Previous studies have found that individuals with aMCI progress to AD at a rate of approximately 5–25% per year^11,12^, while about 16–23% per year reverted from aMCI to normal cognition^13–15^. Therefore, it is crucial to develop prediction criteria that can distinguish individuals with aMCI at imminent risk of conversion to AD dementia from those who will remain stable^16^. In addition, different rates of progression have also been observed among patients with AD^6,17,18^. The rate of disease progression has important implications in clinical practice, as it has been shown to be an important factor in determining the prognosis of AD^19^. To date, there have been various neuroimaging studies for predicting AD prognosis at the individual-level^20–24^. However, these were limited by relatively small sample sizes, and the use of less sophisticated imaging methods such as low-field magnetic resonance imaging (MRI). In addition, although several lines of research attempted to predict conversion to AD in aMCI patients, these studies presented problems related to limited prediction accuracy^23–26^.

In this study, we first aimed to develop a new method for measuring AD-specific similarity of cortical atrophy patterns at the individual-level by employing an individual-level machine learning algorithm, and then to demonstrate the potential of this similarity measure in predicting the individual-level prognosis on the AD continuum. Our machine learning method, as previously demonstrated^4^, presents an individual subject classification based on incremental learning for AD diagnosis and prediction for the progression of AD using cortical thickness data. We adopt this method for training the group-level classifier, and then propose a new similarity measure for an individual-level cortical atrophy pattern compared to that of the representative AD patient. This AD-specific atrophy similarity measure represents how similar the cortical atrophy pattern of an individual subject is to that of a representative AD patient defined using a well-defined AD cohort. Specifically, we demonstrated the efficacy of the proposed measure using a large neuroimaging cohort of 869 cognitively normal (CN) individuals and 473 patients with probable AD dementia. We further validated the AD-specific similarity measure using a longitudinal neuroimaging cohort, by comparing this measure between aMCI converters and non-converters and between AD patients with fast and slow degrees of clinical decline. We hypothesize that the proposed individual assessment method is useful not only for determining diagnosis of an individual subject at a given time, but also to predict how likely their future including both progression to AD (a one-year aMCI follow-up validation) and a prognosis of AD (a five-year AD follow-up validation).

## Results

### Demographic and clinical characteristics

The demographics and clinical characteristics of the study participants were presented in Table 1. For the cross-sectional cohort, patients with AD had significantly higher mean age, lower level of education, and more frequent apolipoprotein E (*APOE)* ε4 allele and hypertension than CN individuals. In the longitudinal cohort, aMCI converters had significantly more frequent *APOE* ε4 alleles and lower baseline mini-mental state examination (MMSE) scores than aMCI non-converters. There were no significant demographic differences between AD slow- and fast-decliners.

**Table 1:** Demographic and clinical characteristics of the study participants.

	Subjects for classifier training			Patients with aMCI				Patients with AD			
	CN	AD	*p*-value	Total	Non-converters	Converters	*p*-value	Total	Slow-decliners	Fast-decliners	*p*-value
N	869	473		79	53 (67.1)	26 (32.9)		27	14 (51.9)	13 (48.1)	
Age, y	65.4 (9.0)	73.0 (9.4)	<0.001	69.7 (8.8)	69.1 (9.0)	70.9 (8.6)	0.410	70.4 (7.6)	72.0 (6.6)	68.8 (8.4)	0.276
Age of onset, y	—	68.5 (10.7)	—	67.1 (8.6)	66.3 (8.6)	68.5 (8.4)	0.308	67.2 (7.7)	68.0 (6.9)	66.4 (8.7)	0.595
Women	599 (68.9)	307 (64.9)	0.133	46 (58.2)	29 (54.7)	17 (65.4)	0.366	18 (66.7)	10 (71.4)	8 (61.5)	0.695
Education, y	11.7 (4.9)	9.4 (5.3)	<0.001	12.1 (4.6)	12.1 (4.4)	12.1 (5.0)	0.971	10.3 (5.1)	9.5 (6.2)	11.2 (3.6)	0.392
*APOE* ε4 present*	135 (22.8)	180 (55.6)	<0.001	29 (39.2)	15 (29.4)	14 (60.9)	0.010	4 (66.7)	2 (50.0)	2 (100.0)	0.467
MMSE	28.5 (2.0)	18.2 (5.5)	<0.001	26.4 (2.4)	27.0 (1.9)	25.4 (3.0)	0.015	21.4 (3.1)	21.1 (3.0)	21.7 (3.3)	0.616
Vascular risk factors											
DM	178 (20.5)	112 (23.7)	0.174	35 (44.3)	26 (49.1)	9 (34.6)	0.225	6 (22.2)	5 (35.7)	1 (7.7)	0.165
Hypertension	260 (29.9)	206 (43.6)	<0.001	31 (39.2)	21 (39.6)	10 (38.5)	0.921	14 (51.9)	8 (57.1)	6 (46.2)	0.568
Hyperlipidemia	238 (27.4)	90 (19.0)	0.001	25 (31.6)	19 (35.8)	6 (23.1)	0.251	6 (22.2)	3 (21.4)	3 (23.1)	1.000
History of IHD	110 (12.7)	43 (9.1)	0.049	17 (21.5)	9 (17.0)	8 (30.8)	0.161	2 (7.4)	1 (7.1)	1 (7.7)	1.000
History of stroke	32 (3.7)	26 (5.5)	0.118	2 (2.5)	2 (3.8)	0 (0.0)	0.316	1 (3.7)	1 (7.1)	0 (0.0)	—

Values are mean (SD) or N (%).

Statistical analyses were performed with Chi-square, Fisher’s exact or Student’s *t*-tests.

*APOE genotyping was performed in 916 (68.3%) of the 1,342 subjects for classifier training; 74 (93.7%) of the 79 patients with aMCI; and 6 (22.2%) of the 27 patients with AD, respectively.

Abbreviations: N = number; SD = standard deviation; CN = cognitively normal; AD = Alzheimer’s disease; aMCI = amnestic mild cognitive impairment; *APOE* = apolipoprotein E; MMSE = mini-mental state examination; DM = diabetes mellitus; IHD = ischemic heart disease.

### Group classification performance

**Figure 1:**
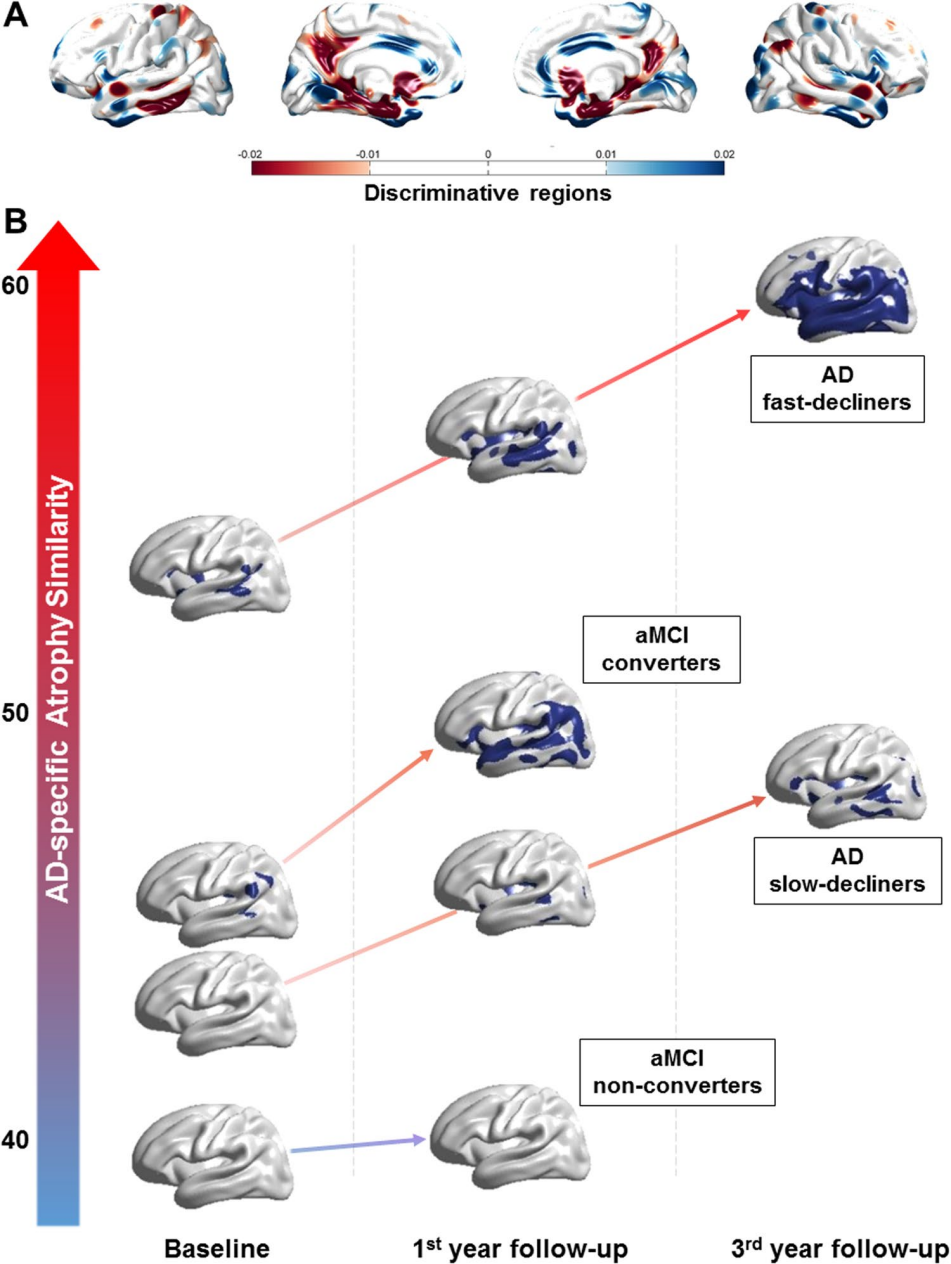
Discriminating features of our classification. (**A**) The discriminating regions of our classification on the atlas surface meshes and (**B**) The discriminative pattern of each patient with aMCI and AD. Color intensities in the figure represent discriminative power in AD classification. aMCI = amnestic mild cognitive impairment; AD = Alzheimer’s disease.

We assessed classification performance using the 10-fold cross-validation procedure on the cross-sectional cohort. Our classifier showed accuracy, sensitivity and specificity values of 91.1%, 83.5%, and 95.2%, respectively, for discriminating AD patients from CN individuals. Figure 1A shows the discriminating regions of our classification on the atlas surface meshes. The colored regions in the figure were determined by the amount of contribution of each vertex to classification. That is, the visualization of the axis that is maximally separating two groups in the linear discriminant analysis (LDA) space represents the contribution of the component to classification^4^. The entorhinal cortex and precuneus were the most discriminative for AD classification, and the lateral temporal lobe and the prefrontal cortex were also discriminative. In addition, we performed the validation of our classification method with the previously proposed method, support vector machine. The discriminative regions for both classifiers were consistent with each other (Supplementary Figure 1).

### Validation of clinical progression in patients with aMCI and AD

Subjects from the longitudinal aMCI and AD cohorts were used to validate the proposed cortical atrophy pattern analysis. Figure 1B visualizes the cortical atrophy patterns of each AD and aMCI patient group over time. The y-axis in the figure represents the AD-specific cortical atrophy similarity measure compared with the representative cross-sectional AD patient cohort used to develop the classifier. In patients with aMCI, non-converters showed no significant discriminative pattern, while converters demonstrated significant discriminative patterns in the inferior parietal lobule at baseline and in the prefrontal, temporal cortices, and inferior parietal lobule at first year follow-up visit, respectively. From the baseline to the third year follow-up visits, AD slow-decliners showed discriminative patterns defined around the prefrontal and temporal cortices, while AD fast-decliners demonstrated significant discriminative patterns in the most of prefrontal, inferior parietal, and temporal cortices.

**Figure 2:**
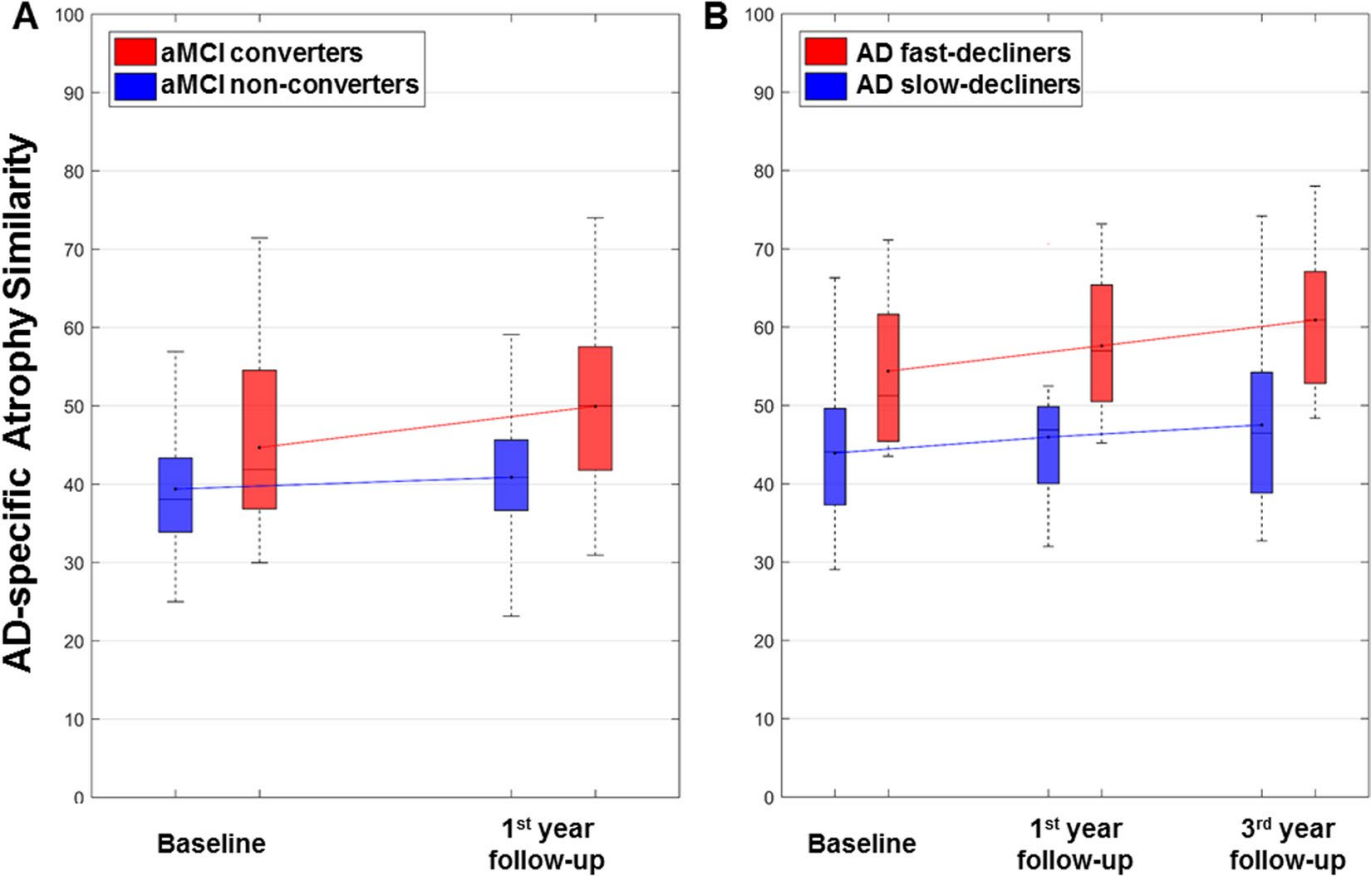
Comparisons of the AD-specific atrophy similarity at baseline and follow-up years: (**A**) non-converters vs. converters in patients with aMCI and (**B**) slow- and fast-decliners in patients with AD. Mixed effects models of the worsening in AD-specific atrophy similarity over time between the classified groups by clinical progression in patients with aMCI and AD showed significant differences between the groups (*p* = 0.027 in aMCI cohort and *p* = 0.029 in AD cohort). aMCI = amnestic mild cognitive impairment; AD = Alzheimer’s disease.

There were significant differences in the AD-specific atrophy similarity measure at both baseline and first year follow-up visits between aMCI converters and non-converters (Fig. 2A). Specifically, converters showed significantly greater increases of the AD-specific atrophy similarity measure over time than did non-converters on a mixed effects model (β = 3.6, standard error [SE] = 1.6, *p* = 0.027). In patients with AD, furthermore, there were significant differences between fast- and slow-decliners in the AD-specific atrophy similarity measure at baseline, first year, and third year follow-up visits (Fig. 2B). AD fast-decliners also showed significantly greater increases of the AD-specific atrophy similarity measure than did slow-decliners on a mixed effects model (β = 2.9, SE = 1.3, *p* = 0.029). Specific details regarding the AD-specific atrophy similarity and neuropsychological performance of both of the longitudinal cohorts by group status can be found in Supplementary Table 1.

Table 2 shows mixed effects models examining how worsening in neuropsychological test performance over time was related to AD-specific atrophy similarity in patients with aMCI and AD. Significant AD-specific atrophy similarity-by-time interactions were obtained for most neuropsychological tests in the two longitudinal cohorts from baseline to year one or three. Specifically, we found AD-specific atrophy similarity by time interactions in both groups for language function, Seoul Neuropsychological Screening Battery-Dementia version (SNSB-D) total score, MMSE, and Clinical Dementia Rating sum of boxes (CDR-SB), while there were significant interactions for only the AD group in attention, memory, frontal/executive function, and Clinical Dementia Rating (CDR).

**Table 2:** Mixed effects models of worsening in the neuropsychological test performances over time by AD-specific atrophy similarity in patients with aMCI and AD.

AD-specific atrophy similarity by time	Patients with aMCI			Patients with AD		
	Estimate	SE	*p*-value	Estimate	SE	*p*-value
Neuropsychological tests						
Attention	−0.018	0.030	0.563	−0.121	0.041	0.003
Language	−0.112	0.036	0.030	−0.344	0.078	<0.001
Visuospatial	−0.032	0.120	0.791	−0.180	0.186	0.338
Memory	−0.370	0.276	0.186	−0.438	0.213	0.044
Frontal/executive	−0.041	0.168	0.809	−0.542	0.169	0.002
SNSB-D total	−0.107	0.350	0.048	−1.604	0.464	0.001
MMSE	−0.064	0.035	0.024	−0.263	0.077	0.001
CDR	0.003	0.003	0.390	0.027	0.010	0.013
CDR-SB	0.042	0.020	0.003	0.145	0.044	0.002

Linear mixed effects model were performed using AD-specific atrophy similarity, time, and the interaction term between AD-specific atrophy similarity and time (AD-specific atrophy similarity by time) as fixed effects and patient as random effect. AD-specific atrophy similarity was computed using w-score based on age and education.

Abbreviations: aMCI = amnestic mild cognitive impairment; AD = Alzheimer’s disease; SE = standard error; SNSB-D = Seoul Neuropsychological Screening Battery-Dementia version; MMSE = mini-mental state examination; CDR = Clinical Dementia Rating; CDR-SB = Clinical Dementia Rating sum of boxes.

## Discussion

In this study, we developed and validated an AD-specific atrophy similarity measure as a novel MRI-based biomarker to provide prospective AD risk prediction on an individual subject level. We found that the AD-specific atrophy similarity measure showed promising results at an individual-level, where it not only supported the early prediction of AD, but also enabled the discrimination of brain and clinical trajectories in patients with AD dementia. The AD-specific atrophy similarity measure, based on cortical thickness analyses we recently developed, was derived from a probabilistic statistical classification model. Our method demonstrated high classification performance in the prediction of AD trajectories and in accurately distinguishing AD patients from normal controls, supporting the discriminative power of our method in both prognosis and diagnosis.

Our conclusion that the AD-specific atrophy similarity measure contributes to prediction of prognosis along the AD continuum is supported by the following observations: (1) in patients with aMCI, converters showed higher AD-specific atrophy similarity than non-converters with increasing scores at baseline and one-year follow-up visits; (2) in patients with AD dementia, fast-decliners also revealed higher AD-specific atrophy similarity than slow-decliners at all visits over a three-year follow-up. More specifically, our findings of the discriminative patterns in patients with aMCI converters and AD fast-decliners were consistent with previous literature findings of predicting AD prognosis which presented changes in the lateral temporal and inferior parietal cortices were related with AD progression^21,22^. Furthermore, significant inverse relationships between the AD-specific atrophy similarity measure and cognitive performance over time were observed in patients with aMCI and AD. Our study therefore provides new insight into both the prediction of aMCI to AD conversion and the prediction of accelerated clinical decline in AD dementia. Further follow-up will allow us to examine whether baseline atrophy similarity measurements can predict the specific time-to-conversion at the individual subject level.

While there have been several recent neuroimaging studies on the prediction of conversion from aMCI to AD dementia, most have exhibited limited prediction accuracy and small sample sizes^23–26^. We investigated the use of the AD-specific atrophy similarity measure as a means to obtain a sensitive and specific biomarker of AD-like spatial patterns of cortical thinning, and of conversion from aMCI to AD within a large cohort. Some recent studies using a cortical thickness-based clustering method demonstrated that AD patients with a parietal-dominant atrophy pattern showed poor performance in neuropsychological tests as well as aggressive rates of progression^6,18^. In comparison, a strength of our study is that different rates of disease progression were investigated by the AD-specific atrophy similarity measure on an individual subject level, and not using cluster or group analyses. Specially, our method has increased statistical power since the AD-specific atrophy similarity measure was derived using machine learning over a large neuroimaging cohort of AD and CN participants. In addition, the current study limited MRI data collection to one scanner with the same scan parameters across waves of data collection, strengthening the consistency of our data and results.

However, some limitations should be considered when interpreting the results. First, pathologic confirmation was not performed in the present study participants. Considering the discrepancy in diagnosis for AD between clinical and neuropathological data^27^, and a certain portion of clinically diagnosed AD patients may show negative amyloid positron emission tomography scan^28^, we cannot exclude the possibility that our classification methods might have been altered by AD-mimicking patients. However, this argument is mitigated to some degree by our previous studies showing that about 90% of clinically diagnosed AD had positive amyloid positron emission tomography scan^29,30^. Second, our classification scheme is based on the assumption that the cortical thickness data could be separated into two categories, such as CN and AD. As some neural network-based methods would be able to handle non-linearity of the feature data, future studies could employ these recently developed deep learning approaches. Third, there is no consensus regarding the time window during which conversion from aMCI to AD must be evaluated, or regarding specific cut-points for defining fast and slow decline in patients with AD. Fourth, two longitudinal cohorts for validation of the AD-specific atrophy similarity had relatively small sample size. Fifth, the proposed classification and AD-specific atrophy similarity measure methods are solely based on the cortical thickness data, and the clinical risk factors and neuropsychological score data were not used. As future works, it would be promising if we could see how those factors can affect the classification performance and atrophy pattern analysis results. Finally, since other classification methods were using various types of feature data with different dataset, it is difficult to compare the classification performance of our method with other methods, directly.

In conclusion, we have developed an AD-specific atrophy similarity measure as a novel MRI-based biomarker. This method provides an innovative approach for enabling the prediction of dementia risk, and for evaluating trajectories along the AD continuum on an individual subject level. Furthermore, while further research is still necessary to validate and further develop the AD-specific similarity measure in other populations, this method will facilitate risk stratification not only for prevention trials but also for personalized therapy.

## Methods

### Study participants

#### Cross-sectional cohort for development of the AD classifier

A total of 536 patients with probable AD dementia and 912 CN individuals who underwent high-resolution 3T brain MRI with 3D volumetric imaging and detailed neuropsychological testing were recruited from the Memory Disorders Clinic of the Samsung Medical Center (from June 2006 to June 2012). The patients with probable AD dementia fulfilled the National Institute of Neurological and Communicative Disorders and Stroke and Alzheimer's Disease and Related Disorders Association (NINCDS-ADRDA) criteria^31^. CN individuals had no history of neurologic or psychiatric disorders, and had normal cognitive function determined using neuropsychological tests (above the 16th percentile for age- and education-matched norms).

We excluded 63 AD patients with any of the following conditions: missing education data (N = 9); unreliable cortical thickness measurements due to head motion, blurring of the MRI, inadequate registration to a standardized stereotaxic space, misclassification of tissue type, or inexact surface extraction (N = 31); or severe white matter hyperintensities (WMH) defined as deep WMH ≥ 25 mm and periventricular WMH ≥ 10 mm (N = 40). Since study participants could have more than one exclusion condition, the final sample size of AD patients was 473. In addition, out of 912 CN individuals, we excluded 22 participants with incomplete demographic data. From the remaining 890 participants, we excluded 21 participants with unreliable analyses of cortical thickness, yielding 869 CN individuals for analysis in this study.

Laboratory tests were conducted in all participants to rule out other causes of dementia, and included complete blood counts, vitamin B12 and folate levels, a metabolite profile, thyroid function tests, and syphilis serology. Participants were also excluded if they had a cerebral, cerebellar, or brainstem infarction, hemorrhage, tumors, hydrocephalus, or severe head trauma.

#### Longitudinal cohort for validation of the AD-specific atrophy similarity measure

A total of 79 aMCI patients were retrospectively recruited from the Memory Disorders Clinic of the Samsung Medical Center (from August 2007 to December 2010). These aMCI patients had completed at least their first year follow-up visit with the same interview and neuropsychological tests as their baseline evaluation, had undergone high-resolution 3T brain MRI with 3D volumetric imaging, and did not have any critical missing data. Patients were diagnosed with aMCI using the Petersen criteria^32^ with the following modifications, which have been previously described in detail^33^: (1) a subjective cognitive complaint by the patient or his/her caregiver; (2) normal Activities of Daily Living (ADL) score determined clinically and with the instrumental ADL scale; (3) an objective cognitive decline below the 16th percentile (−1.0 standard deviation [SD]) of age- and education-matched norms in at least one of four cognitive domains (language, visuospatial, memory or frontal-executive function) on neuropsychological tests; and (4) absence of dementia. Patients with aMCI were grouped as non-converters (N = 53) if they were diagnosed with aMCI at baseline and remained so during their first year of follow-up, and as converters if they were diagnosed with aMCI at baseline and diagnosed with AD during their first year of follow-up, without reversion to aMCI or CN (N = 26).

We also included 36 patients with AD who participated in the prospective, five-year longitudinal Alzheimer’s Disease and Positron Emission Tomography (ADAPET) study, and were recruited from March 2006 to December 2006. The patients fit the criteria of the Diagnostic and Statistical Manual of Mental Disorders, Fourth Edition^34^ and the NINCDS-ADRDA criteria for probable AD^31^. The enrolled patients were eligible if they had early-stage dementia with a CDR score of 0.5 or 1, were cooperative candidates for this longitudinal study, and had a caregiver. None had a family history suggestive of an autosomal dominant disease. Of 36 patients with AD, 27 patients who completed the third year of evaluation were enrolled in the current study. The assessment procedure of the participants has been described in detail elsewhere^35,36^. Patients with AD were grouped as fast-decliners (N = 13) if their CDR-SB score increased more than five points during the three year follow-up; otherwise, they were labeled as slow-decliners (N = 14).

#### Standard protocol approvals, registrations, and patient consents

We obtained written informed consent from each patient. This study was approved by the Institutional Review Board at the Samsung Medical Center. In addition, all methods were carried out in accordance with the approved guidelines.

#### Neuropsychological tests

All participants underwent a standardized neuropsychological battery, the Seoul Neuropsychological Screening Battery (SNSB), which is described in detail elsewhere^37^. The SNSB consists of tests for verbal and visual memory, attention, language, praxis, four elements of Gerstmann syndrome, visuospatial function, frontal/executive function, the MMSE, the CDR, and the CDR-SB. From the SNSB results, we calculated the SNSB-D score in attention, language, visuospatial, memory, and frontal/executive domains, as previously described^37,38^.

#### Image acquisition and preprocessing

3D T1-weighted Turbo Field Echo MRI images were acquired from all participants in this study using the Philips 3T Achieva MRI scanner with the same imaging parameters (sagittal slice thickness 1.0 mm, over contiguous slice acquisition with 50% overlap; no gap; repetition time 9.9 ms; echo time 4.6 ms; flip angle 8°; and matrix size 240 × 240 reconstructed to 480 × 480 over a 240 mm field of view).

**Figure 3:**
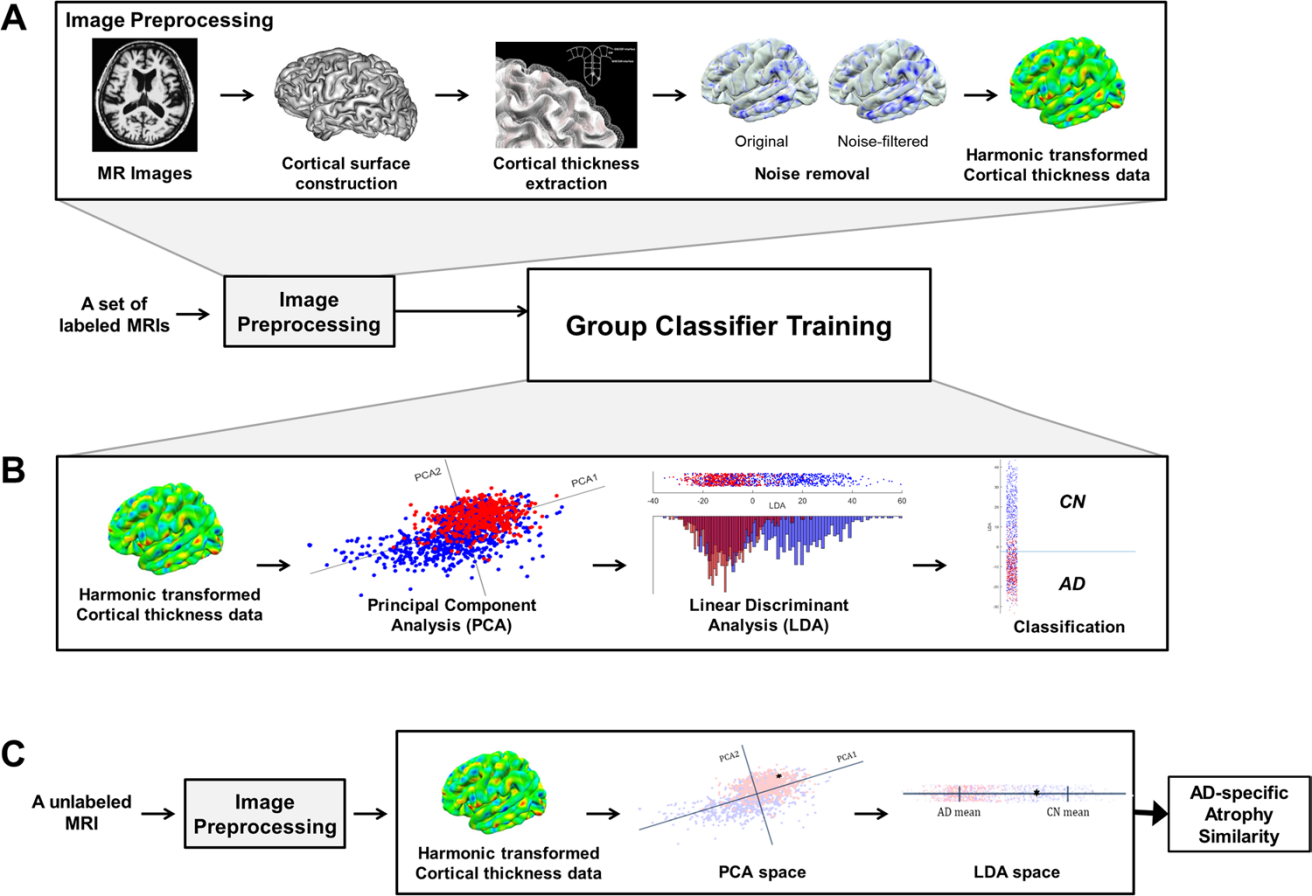
Overview of the proposed method. (**A**) Image preprocessing; (**B**) Group classifier training; and (**C**) AD-specific pattern similarity computation. AD = Alzheimer’s disease.

For each subject, we performed image preprocessing using FreeSurfer 5.1.0 (Athinoula A. Martinos Center at the Massachusetts General Hospital, Harvard Medical School; http://surfer.nmr.mgh.harvard.edu/). Figure 3A shows the overview of our image preprocessing method. We first constructed the outer and inner cortical surface meshes from the MR volume of each subject. The two meshes are isomorphic with the same vertices and connectivity because the outer surface is constructed by deforming the inner surface. In order to establish inter-subject correspondence, we resampled each subject’s cortical surface to 40,962 vertices for each hemisphere using the previously proposed method^4^.

For removing noise in the cortical thickness data, we employed the manifold harmonic transform (MHT) to map the cortical thickness from the surface onto the frequency domain^39,40^. The MHT regarded high frequency components of the transformed cortical thickness data as noise, and then discarded those components^4^. It enables us to remove noise and reduce the dimensionality of the cortical thickness data by filtering out high frequency components.

#### Cortical atrophy pattern analysis

We analyzed the cortical atrophy pattern for each subject based on the preprocessed cortical thickness data. Specifically, cortical atrophy patterns were quantified using Inbrain^®^, a Korea Food and Drug Administration (KFDA)-cleared software and a registered trademark of MIDAS Information Technology Co., Ltd., which performs fully-automated image analysis of brain structures. The proposed method consists of two steps: training a group classifier (Fig. 3B**)** and computing an AD-specific pattern similarity (Fig. 3C**)**. The noise-filtered cortical thickness data was converted to w-score adjusting for age and education level in order to minimize the effects of them on cortical thickness. For classifier training, we used w-scores as feature vectors and employed principal component analysis (PCA) and LDA^41^. Specifically, we reduced the dimensionality of feature vectors with PCA, and found coordinate axes which maximally separated different groups with LDA. Given feature vectors as input, the classifier was trained by performing PCA and LDA in sequence. We calculated the PCA dimension following the methods of our previous paper^4^.

**Figure 4:**
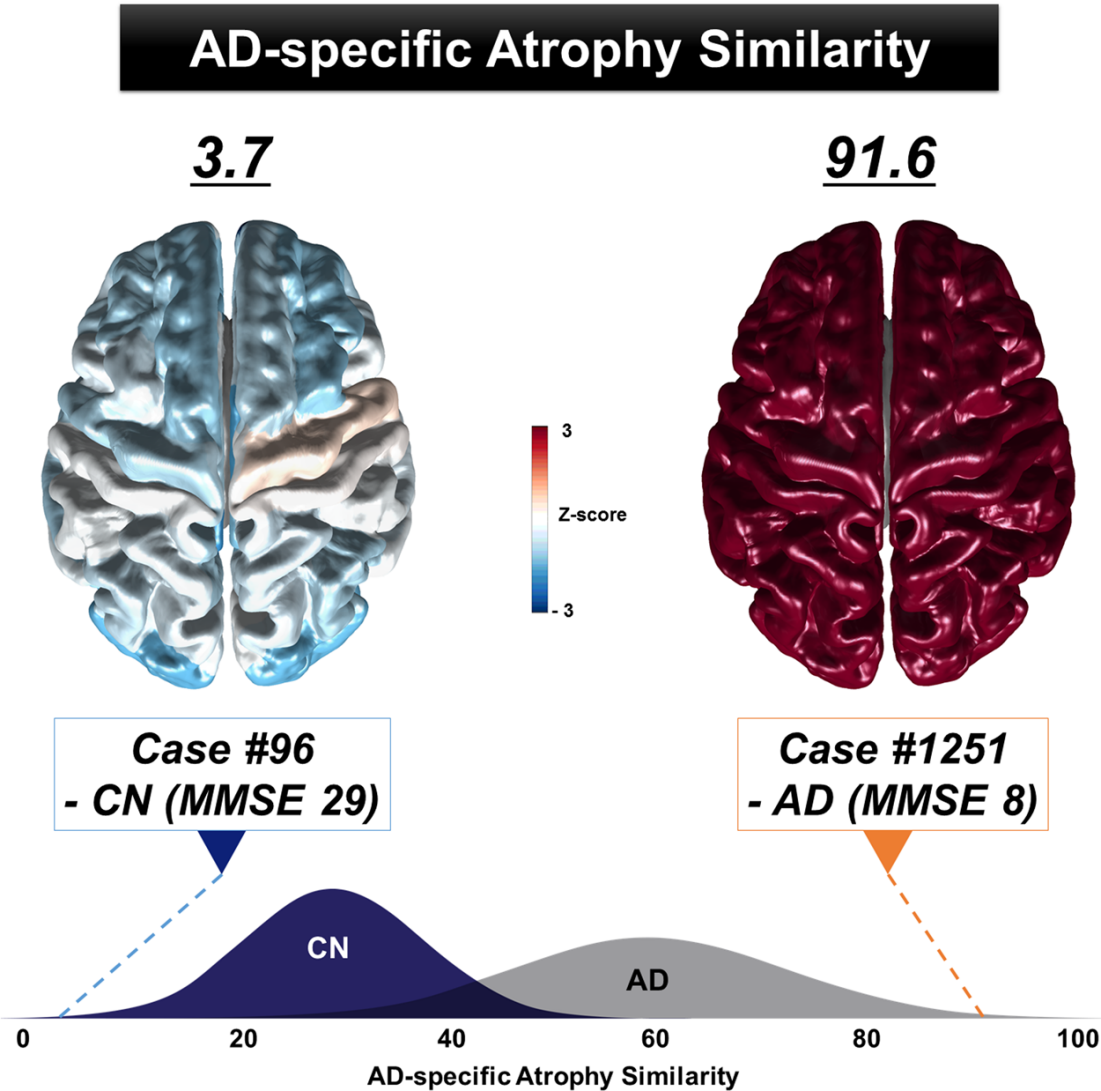
Examples of AD-specific atrophy similarity measure at the individual-level. The AD-specific atrophy similarity scores differed between Case #96 - CN (left, 3.7) and Case #1256 - AD (right, 91.6). The standardized value (Z-score) maps were computed to visualize the AD-specific atrophy similarity. Positive Z-scores (red) indicate that the regions of brain are similar to the AD-specific patterns of atrophy. AD = Alzheimer’s disease; CN = cognitively normal; MMSE = mini-mental state examination.

After training the group classifier, the AD-specific pattern similarity measure was then calculated on an individual subject basis. As shown in Figure 3C, the noise-filtered cortical thickness data of an individual subject was transformed to PCA space using the pre-trained PCA axes. Similarly, the feature vector in PCA space was also mapped onto a single point in LDA space using the pre-trained LDA matrix. Finally, we measured the AD-specific similarity of the cortical atrophy pattern for an individual subject based on the distance between each subject’s mapped point and the mean value of the AD group in LDA space. A higher AD-specific atrophy similarity measure indicates that a subject’s brain atrophy pattern is more similar to the representative pattern of the AD group (Fig. 4).

In order to evaluate group classification performance, we performed a 10-fold cross-validation procedure. We randomly partitioned total participants into two sets of 90% for training and 10% for test. After training the classifier with training data, we assessed the accuracy, sensitivity and specificity of each classification with test data. In addition, we validated the AD-specific atrophy similarity measure in two longitudinal cohorts of patients with aMCI and AD. Specifically, we applied the longitudinal pipelines of FreeSurfer for our longitudinal cohort data. As the FreeSurfer longitudinal pipeline is designed to be unbiased to any particular time, we did not initialize it with information from a specific time point. Instead, a template was created using information from all available time points. This template can be regarded as an initial guess for segmentation of brain regions and surface reconstruction. The longitudinal pipeline consists of three steps^42^: cross-sectional image processing, individual template construction, and longitudinal alignment. According to the template surface, the FreeSurfer-provided *fsaverage* was used, and the smoothing process was applied similarly to the previous work^4^. We manually checked the results of every step and corrected any error that occurred during the surface construction step.

#### Statistical analyses

Continuous variables were presented as means ± SD and were compared using Student’s *t*-test. Categorical variables were compared using the Chi-square test or Fisher’s exact test. To examine how longitudinal changes in neuropsychological test performance over time were associated with AD-specific atrophy similarity in patients with aMCI and AD, we performed linear mixed effects modeling within each cohort using AD-specific atrophy similarity, time, and the interaction term between AD-specific atrophy similarity and time (AD-specific atrophy similarity by time) as fixed effects and patient as a random effect. In addition, to determine whether there are significant differences in the AD-specific similarity over time between the groups by clinical progression in patients with MCI and AD, we also performed linear mixed effects modeling within each cohort using group, time, and the interaction term between group and time (group by time) as fixed effects and patient as a random effect. Statistical significance was set at *p* < 0.05 in two-tailed tests. Statistical analyses were performed using SPSS version 20.0 (SPSS Inc., Chicago, IL, USA).

## Electronic supplementary material

Supplementary Data

## References

[1] Wilson RS (2012). The natural history of cognitive decline in Alzheimer's disease. Psychol Aging.

[2] Singh, V. *et al*. Spatial patterns of cortical thinning in mild cognitive impairment and Alzheimer's disease. *Brain* **129**, 2885–2893. Epub 2006 Sep 2828 (2006). 10.1093/brain/awl256 17008332

[3] Du, A. T. *et al*. Different regional patterns of cortical thinning in Alzheimer's disease and frontotemporal dementia. *Brain* **130**, 1159–1166, Epub 2007 Mar 1112 (2007). 10.1093/brain/awm016 PMC1853284 17353226

[4] Cho Y, Seong JK, Jeong Y, Shin SY (2012). Individual subject classification for Alzheimer's disease based on incremental learning using a spatial frequency representation of cortical thickness data. Neuroimage.

[5] Cuingnet R, Glaunes JA, Chupin M, Benali H, Colliot O (2013). Spatial and Anatomical Regularization of SVM: A General Framework for Neuroimaging Data. IEEE Trans Pattern Anal Mach Intell.

[6] Hwang J (2016). Prediction of Alzheimer's disease pathophysiology based on cortical thickness patterns. Alzheimers Dement (Amst).

[7] Querbes O (2009). Early diagnosis of Alzheimer's disease using cortical thickness: impact of cognitive reserve. Brain.

[8] Davatzikos C, Resnick SM, Wu X, Parmpi P, Clark CM (2008). Individual patient diagnosis of AD and FTD via high-dimensional pattern classification of MRI. Neuroimage.

[9] Sorensen L (2016). Early detection of Alzheimer's disease using MRI hippocampal texture. Hum Brain Mapp.

[10] Shen Q (2011). Volumetric and visual rating of magnetic resonance imaging scans in the diagnosis of amnestic mild cognitive impairment and Alzheimer's disease. Alzheimers Dement.

[11] Grundman M (2004). Mild cognitive impairment can be distinguished from Alzheimer disease and normal aging for clinical trials. Arch Neurol.

[12] Hanninen T, Hallikainen M, Tuomainen S, Vanhanen M, Soininen H (2002). Prevalence of mild cognitive impairment: a population-based study in elderly subjects. Acta Neurol Scand.

[13] Koepsell TD, Monsell SE (2012). Reversion from mild cognitive impairment to normal or near-normal cognition: risk factors and prognosis. Neurology.

[14] Diniz BS, Nunes PV, Yassuda MS, Forlenza OV (2009). Diagnosis of mild cognitive impairment revisited after one year. Preliminary results of a prospective study. Dement Geriatr Cogn Disord.

[15] Ganguli M (2011). Outcomes of mild cognitive impairment by definition: a population study. Arch Neurol.

[16] Petersen RC (2011). Clinical practice. Mild cognitive impairment. N Engl J Med.

[17] Schmidt C (2011). Rapidly progressive Alzheimer disease. Arch Neurol.

[18] Na HK (2016). Malignant progression in parietal-dominant atrophy subtype of Alzheimer's disease occurs independent of onset age. Neurobiol Aging.

[19] Soto ME (2008). Predictive value of rapid decline in mini mental state examination in clinical practice for prognosis in Alzheimer's disease. Dement Geriatr Cogn Disord.

[20] Gaser C, Franke K, Kloppel S, Koutsouleris N, Sauer H (2013). BrainAGE in Mild Cognitive Impaired Patients: Predicting the Conversion to Alzheimer's Disease. PLoS One.

[21] Korolev IO, Symonds LL, Bozoki AC (2016). Predicting Progression from Mild Cognitive Impairment to Alzheimer's Dementia Using Clinical, MRI, and Plasma Biomarkers via Probabilistic Pattern Classification. PLoS One.

[22] Dickerson BC, Wolk DA (2013). Biomarker-based prediction of progression in MCI: Comparison of AD signature and hippocampal volume with spinal fluid amyloid-beta and tau. Front Aging Neurosci.

[23] Cuingnet R (2011). Automatic classification of patients with Alzheimer's disease from structural MRI: a comparison of ten methods using the ADNI database. Neuroimage.

[24] Wei R, Li C, Fogelson N, Li L (2016). Prediction of Conversion from Mild Cognitive Impairment to Alzheimer's Disease Using MRI and Structural Network Features. Front Aging Neurosci.

[25] Risacher SL (2009). Baseline MRI predictors of conversion from MCI to probable AD in the ADNI cohort. Curr Alzheimer Res.

[26] Ardekani, B. A., Bermudez, E., Mubeen, A. M. & Bachman, A. H. Prediction of Incipient Alzheimer's Disease Dementia in Patients with Mild Cognitive Impairment. *J Alzheimers Dis* (2016). 10.3233/JAD-160594 27662309

[27] Beach TG, Monsell SE, Phillips LE, Kukull W (2012). Accuracy of the clinical diagnosis of Alzheimer disease at National Institute on Aging Alzheimer Disease Centers, 2005-2010. J Neuropathol Exp Neurol.

[28] Monsell SE (2015). Characterizing Apolipoprotein E epsilon4 Carriers and Noncarriers With the Clinical Diagnosis of Mild to Moderate Alzheimer Dementia and Minimal beta-Amyloid Peptide Plaques. JAMA Neurol.

[29] Ye BS (2015). Amyloid burden, cerebrovascular disease, brain atrophy, and cognition in cognitively impaired patients. Alzheimers Dement.

[30] Yoon CW (2013). Cognitive deficits of pure subcortical vascular dementia vs. Alzheimer disease: PiB-PET-based study. Neurology.

[31] McKhann G (1984). Clinical diagnosis of Alzheimer's disease: report of the NINCDS-ADRDA Work Group under the auspices of Department of Health and Human Services Task Force on Alzheimer's Disease. Neurology.

[32] Petersen RC (2004). Mild cognitive impairment as a diagnostic entity. J Intern Med.

[33] Seo SW, Cho SS, Park A, Chin J, Na DL (2009). Subcortical vascular versus amnestic mild cognitive impairment: comparison of cerebral glucose metabolism. J Neuroimaging.

[34] Association., A. P. *Diagnostic and statistical manual of mental disorders: DSM-IV*. 4th edn, (American Psychiatric Association, 1994).

[35] Cho H (2013). Longitudinal changes of cortical thickness in early- versus late-onset Alzheimer's disease. Neurobiol Aging.

[36] Cho H (2013). Changes in subcortical structures in early- versus late-onset Alzheimer's disease. Neurobiol Aging.

[37] Ahn, H. J. *et al*. Seoul Neuropsychological Screening Battery-dementia version (SNSB-D): a useful tool for assessing and monitoring cognitive impairments in dementia patients. J Korean Med Sci 25, 1071–1076, 1010.3346/jkms.2010.1025.1077.1071. Epub 2010 Jun 1017 (2010). 10.3346/jkms.2010.25.7.1071 PMC2890886 20592901

[38] Ahn HJ (2011). The cortical neuroanatomy of neuropsychological deficits in mild cognitive impairment and Alzheimer's disease: a surface-based morphometric analysis. Neuropsychologia.

[39] Qiu A, Bitouk D, Miller MI (2006). Smooth functional and structural maps on the neocortex via orthonormal bases of the Laplace-Beltrami operator. IEEE Trans Med Imaging.

[40] Vallet B, Levy B (2008). Spectral geometry processing with manifold harmonics. Computer Graphics Forum.

[41] Belhumeur PN, Hespanha JP, Kriegman DJ (1997). Eigenfaces vs. Fisherfaces: Recognition using class specific linear projection. Ieee Transactions on Pattern Analysis and Machine Intelligence.

[42] Reuter M, Schmansky NJ, Rosas HD, Fischl B (2012). Within-subject template estimation for unbiased longitudinal image analysis. Neuroimage.

