# Supplementary material for: Machine Learning-based Individual Assessment of Cortical Atrophy Pattern in Alzheimer’s Disease Spectrum: Development of the Classifier and Longitudinal Evaluation
Source: Sci Rep. 2018 Mar 7;8:4161. doi: 10.1038/s41598-018-22277-x (PMC5841386; doi:10.1038/s41598-018-22277-x)
Supplement: Supplementary file 1 — Supplementary Data [file 41598_2018_22277_MOESM1_ESM.pdf]

Machine Learning-based Individual Assessment of Cortical Atrophy Pattern in Alzheimer's Disease Spectrum: Development of the Classifier and Longitudinal Evaluation

Jin San Lee,[1-3,*] Changsoo Kim,[4,*] Jeong-Hyeon Shin,[5,*] Hanna Cho,[7] Dae-seock Shin,[8] Nakyoung Kim,[8] Hee Jin Kim,[1,2] Yeshin Kim,[1,2] Samuel N. Lockhart,[9,10] Duk L. Na,[1,2,11] Sang Won Seo,[1,2,11,12,†] Joon-Kyung Seong[5,6,†]

[1]Department of Neurology, Samsung Medical Center, Sungkyunkwan University School of Medicine, Seoul 06351, Korea

[2]Neuroscience Center, Samsung Medical Center 06351, Seoul, Korea

[3]Department of Neurology, Kyung Hee University Hospital, Seoul, Korea

[4]Department of Preventive Medicine, Yonsei University College of Medicine, Seoul, South Korea

[5]Department of Bio-convergence Engineering, [6]School of Biomedical Engineering, Korea University, Seoul, Korea

[7]Department of Neurology, Gangnam Severance Hospital, Yonsei University College of Medicine, Seoul, Korea

[8]MIDAS Information Technology Co., Ltd.

[9]Department of Internal Medicine, Section on Gerontology and Geriatric Medicine, Wake Forest School of Medicine, Winston-Salem, NC, USA

[10]Department of Internal Medicine, Division of Gerontology and Geriatric Medicine, Wake Forest School of Medicine, Winston-Salem, NC 27157, USA

[11]Department of Health Sciences and Technology, [12]Clinical Research Design and Evaluation,

SAIHST, Sungkyunkwan University, Seoul 06351, Korea

* These authors contributed equally as first authors of the paper.

† These authors contributed equally as corresponding authors of the paper.

Corresponding author 1: Joon-Kyung Seong, PhD

School of Biomedical Engineering, Korea University, 145, Anam-ro, Anam-dong 5-ga, Seongbuk-gu, Seoul 02841, Korea.

Tel: +82-2-3290-5660, Fax: +82-2-921-6434, E-mail address: jkseong@korea.ac.kr

Corresponding author 2: Sang Won Seo, MD, PhD

Department of Neurology, Samsung Medical Center, Sungkyunkwan University School of Medicine, 81 Irwon-ro, Kangnam-ku, Seoul 06351, Korea

Tel: +82-2-3410-1233, Fax: +82-2-3410-0052, E-mail address: sangwonseo@empal.com

Supplementary data: Supplementary Table 1 and Supplementary Figure 1

Supplementary Table 1 Comparison of results of the AD-specific atrophy similarity and neuropsychological tests during their years of follow-up in patients with aMCI and AD

	Patients with aMCI			Patients with AD			*p*-value
	Total	Non-converters	Converters	Total	Slow-decliners	Fast-decliners	
**AD-specific atrophy similarity**							
**Baseline**	41.1 (8.7)	39.4 (7.5)	44.7 (10.0)	48.9 (10.6)	43.9 (9.5)	54.4 (9.1)	a, b
**1st year follow-up**	44.0 (9.9)	40.9 (7.6)	49.9 (11.2)	51.5 (11.0)	45.9 (10.0)	57.6 (8.9)	a, b
**3rd year follow-up**				53.9 (12.0)	47.5 (10.6)	60.9 (9.5)	b
**Neuropsychological tests**							
**Baseline**							
**N**	79	53 (67.1)	26 (32.9)	27	14 (51.9)	13 (48.1)	
**Attention**	9.3 (2.3)	9.5 (2.3)	9.0 (2.1)	8.1 (1.4)	7.7 (1.5)	8.6 (1.0)	
**Language**	20.9 (3.5)	21.4 (3.2)	19.8 (4.1)	18.3 (4.7)	18.1 (4.7)	18.5 (4.9)	
**Visuospatial**	30.1 (6.1)	31.3 (4.2)	27.7 (8.4)	27.0 (10.9)	29.6 (9.2)	24.1 (12.1)	
**Memory**	51.3 (16.5)	56.6 (14.9)	40.8 (14.6)	29.4 (11.6)	32.5 (12.4)	26.1 (10.1)	a
**Frontal/executive**	36.7 (10.0)	38.5 (9.2)	33.0 (10.7)	36.5 (11.1)	38.4 (11.9)	34.5 (10.2)	a
**SNSB-D total**	149.7 (26.0)	157.8 (20.6)	133.2 (28.4)	119.4 (28.3)	126.4 (29.4)	111.8 (26.0)	a
**MMSE**	26.4 (2.4)	27.0 (1.9)	25.4 (3.0)	21.4 (3.1)	21.1 (3.0)	21.7 (3.3)	a
**CDR**	0.5 (0.1)	0.5 (0.2)	0.5 (0.0)	0.8 (0.3)	0.8 (0.3)	0.8 (0.3)	a
**CDR-SB**	1.2 (0.8)	1.0 (0.8)	1.6 (0.8)	4.7 (1.8)	4.4 (1.4)	5.0 (2.2)	a
**1st year follow-up**							
**N**	66	43 (65.2)	23 (34.8)	25	13 (52.0)	12 (48.0)	
**Attention**	8.9 (2.1)	9.4 (1.9)	7.9 (2.3)	8.0 (1.9)	7.9 (2.2)	8.1 (1.6)	
**Language**	20.6 (4.0)	21.2 (3.8)	18.8 (4.3)	16.9 (5.4)	17.9 (4.8)	15.8 (6.0)	b
**Visuospatial**	29.2 (7.5)	30.7 (5.6)	25.0 (10.4)	25.4 (11.1)	29.8 (7.6)	20.7 (12.6)	b
**Memory**	49.3 (18.1)	52.7 (17.4)	39.8 (17.5)	28.0 (12.4)	32.5 (12.5)	23.1 (10.7)	b
**Frontal/executive**	37.7 (11.5)	40.4 (9.5)	30.2 (13.9)	34.7 (11.7)	39.4 (8.6)	29.7 (12.9)	a, b
**SNSB-D total**	145.5 (29.9)	152.7 (26.1)	128.6 (32.4)	113.0 (33.1)	127.4 (26.0)	97.4 (33.8)	a, b
**MMSE**	25.3 (3.9)	26.8 (2.5)	22.3 (4.4)	19.9 (4.0)	20.7 (2.8)	19.1 (4.9)	a
**CDR**	0.6 (0.3)	0.5 (0.1)	0.7 (0.4)	0.9 (0.5)	0.8 (0.3)	1.1 (0.5)	a, b
**CDR-SB**	2.1 (1.8)	1.3 (0.9)	3.5 (2.1)	5.7 (2.6)	4.7 (1.8)	6.7 (2.9)	a, b
**3rd year follow-up**							
**N**				27	14 (51.9)	13 (48.1)	
**Attention**				6.4 (2.7)	7.3 (1.8)	5.5 (3.3)	
**Language**				12.9 (7.4)	16.3 (5.6)	9.2 (7.6)	b
**Visuospatial**				17.6 (14.4)	24.5 (11.5)	10.2 (13.9)	b

**Memory**	21.0 (13.5)	28.5 (11.8)	12.9 (10.6)	b
**Frontal/executive**	26.3 (15.4)	35.6 (10.1)	16.2 (13.8)	b
**SNSB-D total**	84.1 (48.7)	112.2 (32.2)	53.9 (45.8)	b
**MMSE**	15.9 (6.5)	18.9 (4.5)	12.5 (6.9)	b
**CDR**	1.6 (0.8)	1.0 (0.4)	2.2 (0.6)	b
**CDR-SB**	9.3 (4.2)	6.1 (2.4)	12.6 (2.9)	b

Values are mean (SD) or N (%). Age and education were included as covariates for analyses of covariance, in comparison of the AD-specific atrophy similarity and neuropsychological test performances among the groups.

[a]$p < 0.05$ with Bonferroni's post hoc analyses comparing non-converters and converters in patients with aMCI.

[b]$p < 0.05$ with Bonferroni's post hoc analyses comparing slow- and fast-decliners in patients with AD.

Abbreviations: N = number; SD = standard deviation; AD = Alzheimer's disease; aMCI = amnestic mild cognitive impairment; SNSB-D = Seoul Neuropsychological Screening Battery-Dementia version; MMSE = mini-mental state examination; CDR = Clinical Dementia Rating; CDR-SB = Clinical Dementia Rating sum of boxes.

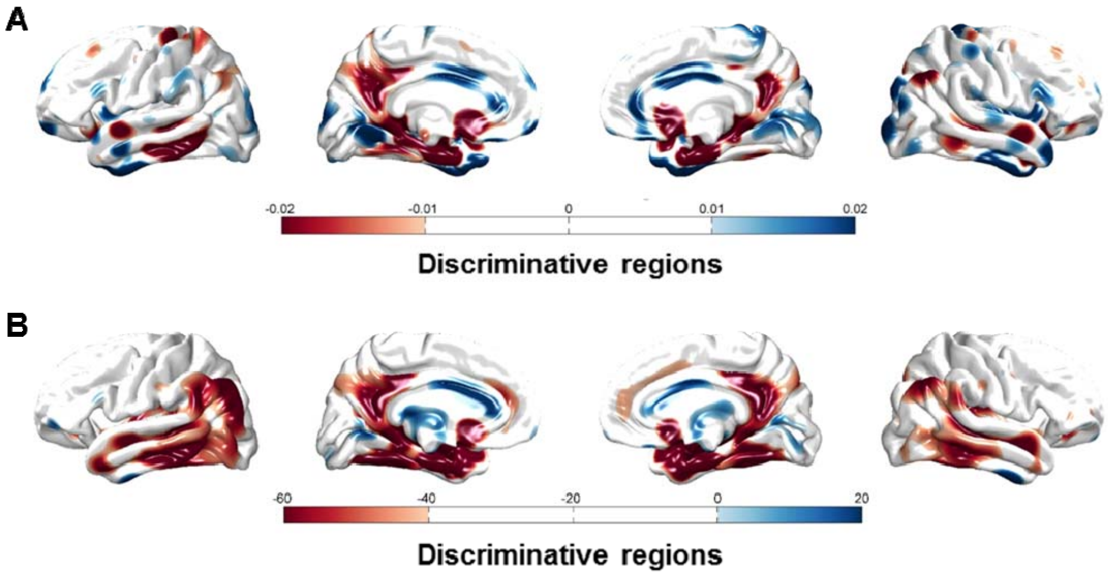

Supplementary Figure 1 Comparison of the discriminative regions between the (A) PCA/LDA- and (B) SVM-based methods. Color intensities in the figure represent discriminative power in AD classification. In the case of the linear SVM, the value of the *i*-th component of the vector *v* that is orthogonal to the separating hyperplane represents the contribution of the component to classification. The discriminative regions for both classifiers are consistent with each other.

PCA = principal component analysis; LDA = linear discriminant analysis; SVM = support vector machine; AD = Alzheimer's disease.
